# “Tell me about”: a logbook of teachers’ changes from face-to-face to distance mathematics education

**DOI:** 10.1007/s10649-021-10108-2

**Published:** 2021-10-15

**Authors:** Giovanna Albano, Samuele Antonini, Cristina Coppola, Umberto Dello Iacono, Anna Pierri

**Affiliations:** 1 Department of Computer Engineering, Electrical Engineering and Applied Mathematics, University of Salerno, Fisciano (SA), Italy; 2 Department of Mathematics and Computer Science, University of Florence, Florence, Italy; 3 Department of Mathematics, University of Salerno, Fisciano (SA), Italy; 4 Department of Mathematics and Physics, University of Campania “L. Vanvitelli”, Caserta, Italy

**Keywords:** Pandemic emergency, Distance education, E-learning tetrahedron model, Attitude

## Abstract

In 2020, the emergency due to the COVID-19 pandemic brought a drastic and sudden change in teaching practices, from the physical space of the classrooms to the virtual space of an e-environment. In this paper, through a qualitative analysis of 44 collected essays composed by Italian mathematics teachers from primary school to undergraduate level during the spring of 2020, we investigate how the Italian teachers perceived the changes due to the unexpected transition from a face-to-face setting to distance education. The analysis is carried out through a double theoretical lens, one concerning the whole didactic system where the knowledge at stake is mathematics and the other regarding affective aspects. The integration of the two theoretical perspectives allows us to identify key elements and their relations in the teachers’ narratives and to analyze how teachers have experienced and perceived the dramatic, drastic, and sudden change. The analysis shows the process going from the disruption of the educational setting to the teachers’ discovery of key aspects of the didactic system including the teacher’s roles, a reflection on mathematics and its teaching, and the attempt to reconstruct the didactic system in a new way.

Not just everything But at least something will be fine!

(A mathematics secondary school teacher)

## Introduction

This study aims to explore how Italian mathematics teachers managed their teaching activities in the context of a total lockdown imposed as part of the government response to the COVID-19 pandemic. The lockdown was decreed in different parts of Italy at different times between the end of February and the beginning of March 2020, a timeframe in which activities in almost all sectors were interrupted and citizens were forced to stay at home. Italy’s education systems did not constitute an exception to this norm: schools and universities were suddenly closed, and teachers and learners shifted from the usual face-to-face to distance education. As a consequence, the learning process moved from the physical space of the classrooms to the virtual space of an e-environment. The reorganization of didactics in the schools has not been structured. The responsibility of such reorganization has been in the charge of the didactic managers and the teachers. Each teacher or each institution created their own e-environment, choosing an online teaching platform with communication and collaboration facilities and eventually with software for specific domains. New teaching settings required the teachers to engage with new designs of their teaching processes, impacting also on the affective aspects of such processes.

Our study took place about a month after the forced closure of schools, at a time that education was at full distance with no prospects for a return to the classroom.

The situation in which our study moves is not like a typical distance setting before the pandemic in which participants do not know each other or they are located across large geographic areas. In our context, the pandemic forced teachers and students to move to the new online distance settings. As this move happened as a consequence of government decisions, teachers and learners had very limited prior technological and/or methodological preparation that foresaw the new settings.

In this frame, we are interested in exploring how teachers of mathematics perceived and responded to the abrupt transition from face-to-face to distance education. In particular, we look at the teachers’ attitude towards mathematics teaching within the new educational system. We base our study on the integration of two lenses: the e-learning tetrahedron model (Albano, 2017) and the teachers’ attitude model towards mathematics and its teaching (Coppola et al., 2012). Offering a systemic view of distance or blended didactical environment for mathematics teaching, the e-learning tetrahedron model identifies four main actors—the Student, the Author, the Tutor, and the Mathematics—as vertices of the tetrahedron and main actors of the system. These actors move within a global technological environment that didactically is intentionally used for mathematics teaching. The teachers’ attitude model towards mathematics and its teaching refers in turn to three attitudinal aspects, namely, the emotional disposition, the view, and the perceived competence.

It is by integrating these two lenses that this article intends to read the teachers’ attitudes during the movements within the didactical system. To this end, we conducted a survey among Italian mathematics teachers from primary school to the undergraduate level. We selected a narrative approach, asking teachers to tell about their experience, focusing on affective aspects, and reflecting on their role as teachers during this time of sudden change.

## Conceptual frameworks

To date, literature concerning e-learning mathematics education is focused mainly on research in blended settings (online and face-to-face) (Borba & Llinares, 2012; Silverman & Hoyos, 2018; Engelbrecht et al., 2020). So as far as work on distance settings, this literature stream is mainly concerned with massive open online courses (MOOCs) (see, for example, Borba et al., 2016; Taranto & Arzarello, 2019), in which there is only a virtual class or distance education practices for communities of learners located across large geographic areas with minimal opportunities for interaction (for example, see Lowrie & Jorgensen, 2012).

Similarly, the literature regarding the role of affective factors (beliefs, emotions, attitude) in mathematics teaching and learning is widely consolidated (Batchelor et al., 2019; Schukajlow et al., 2017; Zan et al., 2006). There are however no studies of affect in distance learning settings for mathematics. Some studies focused on the management of emotions and affectivity in intelligent tutoring systems, where the detection of the emotional affective feeling of a learner is exploited to build a suitable and personalized support to stimulate attention and learning.^1^ The unprecedented exceptionality of a crisis like the pandemic—the first event of its kind in almost a century—explains why there is no literature about mathematics education in distance learning or about mathematics teachers’ affect concerning an anomalous educational context such as the one we are studying.

Because of the context frame of our study, we think that two different theoretical frameworks can offer powerful tools for the analysis we want to perform. The first one is the *e-learning tetrahedron model* (Albano, 2017), which offers a systemic view of changes in an educational system and may allow us to frame teachers’ reflections on their own roles within the “new” educational system. The second one is the *teachers’ attitude model* (Coppola et al., 2012), founded on the attitude model of Di Martino and Zan (2010), which allows us to analyze teachers’ attitudes along the changes that occurred within this “new” educational system. In the following, we present the two theoretical frameworks. We believe that the tetrahedron model can allow us to describe important elements of the teachers’ perception of the changes. At the same time, we add a focus on the affective factors, which are not considered by the tetrahedron model yet are the primary objects of the teachers’ attitude model.

### The tetrahedron model

To model the educational system, we use the e-learning tetrahedron (Albano, 2017), which arises within blended education research and takes into account the dynamics of a (non-virtual) classroom. Albano’s model can be considered as an extension of the didactics triangle (Chevallard, 1985), bearing in mind the huge introduction and use of technology features and more finely articulating the teacher’s role.

**Fig. 1:**
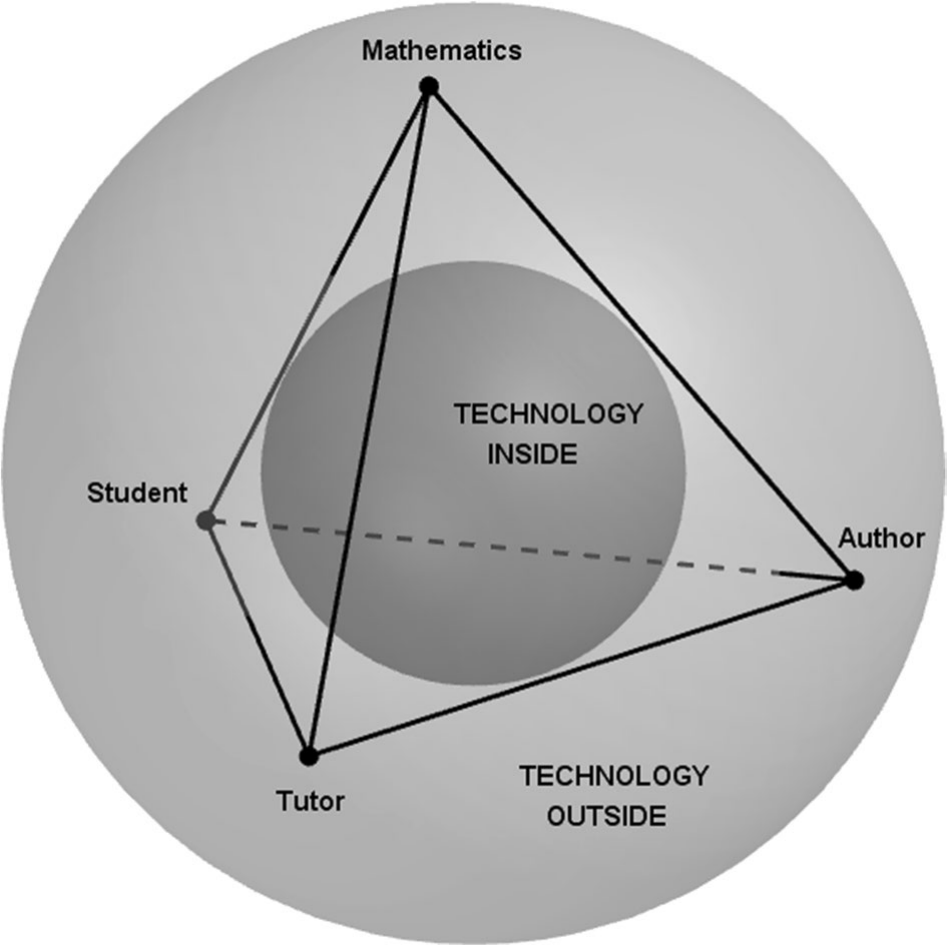
The e-learning tetrahedron model

The actors of the didactics system have been modeled as tetrahedron vertices (Fig. 1) and are the following:

- The Student, who is the one addressed by the teaching process
- The Author, who is a collection of experts with various professional skills (technology, pedagogy, mathematics education), collaborating and looking at the educational project from different perspectives for a nontrivial and effective exploitation of technology
- The Tutor, who takes care of scaffolding and fostering the student’s learning process
- The Mathematics, that is, the knowledge to be taught/learnt.

Technology is considered inside and outside the didactic system: on one hand, we are immersed in a technology-connected world (outside the tetrahedron); on the other hand, the intentional use of technology for teaching and learning poses the technology within the didactic system (inside the tetrahedron). Looking at the faces of the tetrahedron can give a view on various facets of the educational process. The face Author–Mathematics–Student concerns a context where the Student can autonomously interact with the Mathematics, starting from a didactical transposition by the Author or constructing herself some new resources, so acting as an author. The face Mathematics–Student–Tutor highlights the mediation between students and knowledge to be constructed, realized by interactions and communication with an expert. The face Author–Mathematics–Tutor focuses on the design and validation of the activities in the e-environment, realized by the collaboration among people who plan and people who interact with the students. The face Author–Student–Tutor refers to bridging action of the Tutor between the Author and the Student, as a mediator in one direction and as a feedback collector in the converse direction.

A further feature of the tetrahedron model is the dynamicity of its vertices, intended as positions/roles that any actor of the didactic system can play. This makes it possible to design and analyze didactical situations where the student can be in charge of some didactical functions (Albano, 2017), such as creating didactic materials (i.e., Author) or being a tutor among peer or younger students (i.e., Tutor), which adheres to the e-learning promise of putting the student at the center of the learning process, not only as someone who learns (Chevallard & Ladage, 2008). We assume that this dynamicity can also concern the teacher, especially in the case of moving from a face-to-face setting to a technology-based distance setting.

### The teachers’ attitude model

Recent developments in mathematics education research raised growing awareness on the importance of affective aspects. The field of affect developed over the past three decades, in recognition of the relevance that such aspects held in studying the complexity of the process of teaching and learning mathematics (Di Martino, 2019; Di Martino & Zan, 2015; Hannula et al., 2018; McLeod, 1992). The relevance of this issue is strongly true for teachers, as noted by Zembylas (2005, p.467):

Teacher knowledge is located in ‘the lived lives of teachers, in the values, beliefs, and deep convictions enacted in practice, in the social context that encloses such practices, and in the social relationship that enliven the teaching and learning encounter’ (Britzman, 1991, p. 50). These values, beliefs and emotions come into play as teachers make decisions, act and reflect on the different purposes, methods and meanings of teaching.

Moreover, many studies showed how what teachers believe and feel has a clear influence on what students believe and feel (e.g., Tsamir & Tirosh, 2009). While this is true in general, it is even more important, in our analysis, to use the lens of affect research to investigate what is happening for teachers during the pandemic, when everyone had a very strong critical experience, approaching what Bruner (1990) calls *turning points*. To analyze teachers’ affect, it is necessary to consider not only their attitude towards mathematics but also towards its teaching, as highlighted in many studies in mathematics education (for instance, Relich et al., 1994).

More particularly, we are interested in the teachers’ perception of their experience during the first lockdown period. As we will see below, we accessed these experiences through teachers’ narratives, which focused on their awareness of different aspects of the lockdown situation as well as their awareness of how they are living the changes brought forward by the onset of state-sanctioned isolation and remote working. It is precisely to highlight these latter points that we opted to combine the affective aspects in the teaching and learning process within the didactic system described by the tetrahedron model, exploiting the three-dimensional model of attitude (TMA) of Di Martino and Zan (2010) and the teachers’ attitude towards mathematics and its teaching (TAMT) model of Coppola et al. (2012). The TMA comes from a grounded definition of attitude that is a multidimensional characterization of the construct strictly linked to students’ experience collected in a large sample of autobiographical essays. The TAMT is an extension of the TMA model coming from a study about attitude towards mathematics and its teaching in prospective teachers, aimed to make them aware of the influence of affective aspects on the teaching and learning process. In addition to the three TMA dimensions—emotional disposition towards mathematics, view of mathematics, and perceived competence towards mathematics—the TAMT model also considers the teachers’ emotional disposition towards mathematics teaching, their views of mathematics teaching, and perceived competence towards mathematics teaching. The resulting six dimensions are strictly intertwined with each other, in particular regarding how a positive or negative relationship with mathematics can influence the way of teaching, in terms of view, emotions, and perceived competence.

As anticipated in the Introduction, we aim to investigate the teachers’ perceptions of the changes due to the transition from a face-to-face setting to distance education within the new educational system. The tetrahedron model and the teachers’ attitude model fit very well with our aim to investigate the changes in teaching/learning processes starting from teachers’ perceptions. Through this theoretical frame, we will consider the changes in the teachers’ relationships with the vertices of the tetrahedron, paying particular attention to the linked affective factors.

## Methodology

At the end of March 2020, a month from the onset of total lockdown in Italy, when every face-to-face activity was suspended across the country, we distributed a call for essays among Italian teachers from various grade levels, since teachers of every level were involved in the shift to distance education. We conducted a qualitative study using a narrative approach. We set an online form with one open question, given in Italian, with the following instructions (in the English translation):

We ask you to write an essay entitled ‘Teaching/learning in the days of the coronavirus. From face-to-face to distance education. Logbook of a change’. You can deepen the aspects you consider most important, whether they are cognitive and methodological or affective (emotions - fears, enthusiasm, etc.; beliefs and expectations - about effectiveness, teacher-student ‘contact’, students’ involvement, etc.) or metacognitive (reflections). Please, give more details also on how your choices and beliefs about the various aspects considered have changed as your distance learning experience has progressed. The essay can be of varying length, organized as you prefer (e.g., a single text or a daily journal).

We disseminated the form link by means of emails sent to all teachers who are members of the Italian Association for Research in Mathematics Education and to teachers who are part of local communities known to the authors of this paper. The teachers joining the call had about 1 month to submit the essay.

In line with established methodologies of narrative data collection (Kaasila, 2007), we preferred essays that described stories in which the narrator selected the most significant aspect independently, without answering others’ questions or focusing on aspects they deemed to be irrelevant (Di Martino & Zan, 2010). Moreover, as Kaasila underlines, we can focus not only on narrators’ experiences but also on how they describe such experiences. Indeed, Connelly and Clandinin (1990, p. 2) state that: “The main claim for the use of narrative in educational research is that humans are storytelling organisms who, individually and socially, lead storied lives. The study of narrative, therefore, is the study of the ways humans experience the world.”

A priori we chose to frame the collected data from a systemic point of view, using the tetrahedron model, by taking into account the affective aspects involved, using the teachers’ attitude model. The qualitative analysis of the essays showed that the changes described by the teachers can be interpreted by us as “movements” in the tetrahedron, where the affective aspects assume an important role.

We refer to “movement towards a vertex” when the teacher assumes the role, respectively, of Student, Author, Tutor, and of a mathematics scholar who reflects on Mathematics and on its teaching.

In this perspective, we highlight how the various teachers’ movements emerge from their narratives, as follows:

- *The teacher moves towards the Student*
  The teacher describes herself as a student, that is, one who must/wants to learn something new. In our context, we look for excerpts showing the teacher’s need/wish to learn (e.g., to learn how to use the new tools, how to manage the new situation, how to use the technological tools).
- *The teacher moves towards the Author*
  The teacher describes herself as someone who feels the need to take part in the process of designing resources, tasks, and activities and setting up teaching/learning situations suitable for given didactical objectives. Here, we look for excerpts wherein the teacher refers to herself as being in charge of the design for learning.
- *The teacher moves towards the Tutor*
  The teacher tells about the need of building a new relationship, less asymmetrical, with her students both at affective and cognitive levels. We look for sentences revealing more and different interactions and attention to the students.
- *The teacher moves towards the Mathematics*
  The teacher tells about the need for a reflection on Mathematics with respect to the teaching/learning process. Here, we look for the excerpts from which this reflection emerges.

For every movement towards a vertex, we observed related changes of teachers’ attitudes that show us why and how these movements happen. More precisely, we observed the attitude towards the “new” processes of mathematics teaching and learning, by looking for emotional disposition, view, and perceived competence coming out from the teachers’ words. Table 1 gives examples of each kind of move using bold type to highlight the wording that led us to identify the excerpt^2^ in the given category. In the first excerpt, we can see that the teacher describes herself as someone who sets out to study and face new challenges. The second excerpt shows a teacher who recognizes the need of being engaged in the didactic design. The teacher in the third excerpt makes evident the change of her relationship with the students, no longer focused only on teaching. The fourth excerpt reveals a teacher who reflects on the nature of mathematics and on new ways of teaching it.

**Table 1:** Examples of essays’ analysis (the bold words shed light to the teacher’s movement)

Teacher’s movements towards	Excerpts﻿
The **Student**	***I started studying*** *how to carry out distance learning. After all, the peculiarity of the teachers is* ***to be “long-life students”, to maintain (hopefully always) the desire to learn*** *and not to retreat in the face of challenges*
The** Author**	*Distance education needs* ***a preliminary phase of didactic design through the selection of contents, the identification of objectives*** *and the contextualization of the didactic unit within the disciplinary program.* ***A work of selection and organization of materials to be used***, *of activities to be proposed for education and curricular disciplines carried out in synchronous and asynchronous way*
The **Tutor**	*What is changed is that now it is no longer a question of homework or teaching,* ***it is about being an adult reference and less a teacher***. *Having an appointment every day helps to sustain this situation made of fear, sometimes of flimsy internet connections, of crowded rooms, of embarrassment in showing one’s home intimacy and nostalgia of the classmates*
The **Mathematics**	*I am experiencing that* ***these didactic goals are achievable without complicating life with unnecessary algebraic “baroque” manipulations*** *(for example solving equations by means of 15 simplification steps before arriving at the “final” equation itself …). The students are reacting well. Better than they did pre-coronavirus*

## Data analysis and results

We collected 44 essays throughout Italy, eight from primary (1st to 5th grade) teachers (indicated by PT#), ten from teachers of the 6th to 8th grade (indicated by MT#), 17 from high school (9th to 13th grade) teachers (indicated by ST#), and nine from university teachers (indicated by UT#). The excerpts presented in the analysis that follows have been chosen as representative of the various aspects that are the focus of our observation and analysis. For every excerpt, we include the original Italian quotations in square brackets.

From an emotional standpoint, the incipit of essays PT2, PT6, MT3, MT6, ST5, ST13, ST14, and ST16 is very strong, recalling the moment when everything changed. Many teachers begin their narratives by telling about “that” particular day, the weather or the sensations, and perceptions they felt. A very detailed emotional contextualization that shows how that was experienced as a “watershed,” a *turning point* (Bruner, 1990) after which nothing has been as before:

*That Friday at school, the last before Carnival holidays, was a strange day. And I don’t know why, but I didn’t want to go home: after class, I would like to stay at school*﻿. (ST13)

[Quel venerdì di scuola, l’ultimo prima delle vacanze di Carnevale, era una giornata strana. E non so perché, ma non volevo tornare a casa: finite le lezioni, volevo rimanere a scuola.]

*It is a Monday in the beginning of March in a particularly dry winter, in which the sunny days have followed one another with a stable and pleasant trend. There is in the air, now close to the equinoctial transition, a flash of estrangement, which immediately reverberates in the eyes of the students. [...] Nobody could imagine what was going to happen. [...] That’s why, this morning I feel vulnerable to those glances. […] Three days after the dive into surreality: we sink abruptly, like Carroll’s Alice, into a space with an unknown and deforming topology*. (ST14)

[È un lunedì dell’inizio di marzo di un inverno particolarmente secco, in cui le giornate di sole si sono susseguite con andamento stabile e gradevole. C’è nell’aria, oramai vicina alla transizione equinoziale, un guizzo di straniamento, che riverbera immediato negli occhi dei ragazzi. [...] Nessuno immaginava quello che stava per succedere. [...] Ecco perché, questa mattina mi sento vulnerabile davanti a quegli sguardi [...] Tre giorni dopo il tuffo nella surrealtà: sprofondiamo bruscamente, come la Alice di Carroll, in un spazio dalla topologia sconosciuta e deformante.]

*50 days have passed since the morning when the bell stopped ringing. I still feel the echo of it*. (ST16)

[Sono ormai trascorsi 50 giorni dalla mattina in cui la campanella ha smesso di suonare. Ne sento ancora l’eco.]

Only after such touching prose, do the teachers continue narrating what happened to their teaching process in the new distance education setting.

### The teacher moves towards the Student

The new situation led the teachers to move towards the Student, in the sense that they narrate the experience of “becoming Students.” This movement takes place at two levels. On the one hand, the teacher becomes a Student because she feels the need of an educational training that will allow her to acquire skills in managing the new situation from a teaching point of view. On the other hand, the teacher becomes a Student to acquire digital skills that she is forced to use in distance education.

The movement seems to be characterized by different attitudes (positive, negative, or changing) towards mathematics teaching in the new situation, with the model’s dimensions strongly intertwined. As a first reaction to the new situation, the writers of essays PT2, PT6, MT2, and ST16 claim to be uncomfortable. This emotional disposition is often linked to the fear of not being able to preserve the vision of mathematics and of teaching mathematics in the transition to distance education:

*At the beginning I was quite shocked: I was not prepared and I felt it to be very distant from my usual way of teaching ... Not being able to interact with the children, hear their opinions and listen to their suggestions, PLAYING AS A TEAM WITH THEM...IT MADE ME SAD AND IT BLOCKED ME*.^3^ (PT2)

[Inizialmente mi ha abbastanza sconvolto: non ero preparata e la sentivo lontana dal mio modo di insegnare… Non poter interagire con i bambini, sentire le loro opinioni ed ascoltare i loro suggerimenti, FARE GIOCO DI SQUADRA...MI RATTRISTAVA E BLOCCAVA.]

The teachers PT1, PT2, PT3, PT5, PT6, ST3, ST13, ST16, and UT1 declare a feeling of sadness for their prediction of losing positive emotions without being face-to-face in the schools:

*I will never succeed ... in distance education it is impossible! I will miss the best! …* (PT1)

[Non riuscirò mai …con la didattica a distanza è impossibile! Mi perdo il meglio!]

For some of those (PT5, PT6, ST3) who claim to feel this way, the view of mathematics teaching emerging from the essays is as something that passes strongly through the relationships that are established in the classroom and therefore as something that they find difficult to rethink in the distance setting.”

*Obviously the Distance Education caught me ‘unprepared’. Due to my character and the characteristics of my students, my teaching is very much based on relationship, empathy, emotion and not least on physicality, very often theatrical, in the classroom. *(ST3)

[Ovviamente la DAD mi ha colto ‘impreparato’. Per mio carattere e per le caratteristiche dei miei alunni, il mio insegnamento si basa moltissimo sul rapporto, sull’empatia, sull’emozione e non da ultimo sulla fisicità, molto spesso anche teatrale, in classe.]

In PT5, MT7, and MT11 essays, the negative emotions of the initial bewilderment are linked to a sense of inadequacy, therefore to a very low perceived competence towards teaching in the new situation:

*I was asked about distance learning while I could only ever describe my discomfort and my inadequacy, because that’s what I feel*. (MT7)

[Mi si chiedeva della didattica a distanza mentre io invece riuscivo a descrivere sempre e solo il mio disagio e la mia inadeguatezza, perché in fondo è proprio questo quello che provo.]

However, essays PT1, PT2, MT2, MT9, MT11, ST3, ST2, and ST16 reveal that, after the initial emotion of discouragement, there is a positive change in attitude. We believe this change is linked to the rethinking of one’s own vision and beliefs, to the desire to get back into the game as students, accompanied by positive emotions of challenge.

*I started ‘studying’ how to carry out distance learning. After all, the peculiarity of the teachers is to be ‘lifelong students’, to maintain (hopefully always) the desire to learn and not to retreat in the face of challenges*. (ST2)

[Ho cominciato a ‘studiare’ come poter fare didattica a distanza. In fin dei conti la peculiarità dei docenti è quella di essere ‘studenti a vita’, di mantenere (si spera sempre) la voglia di imparare e di non arretrare di fronte alle sfide.]

ST16 explicitly states:

*Even my vision of distance education has changed during this period, due to the continuous interactions with the pupils, almost 24 h a day!* (ST16)

[Anche la mia visione di didattica a distanza ha avuto, in questo periodo, un mutamento, grazie anche alle continue interazioni con gli alunni, quasi 24 ore su 24!]

Indeed, as suggested in the last excerpt, some teachers (PT1, PT2, PT5, MT7, ST3, ST13) note that the incentive for this change comes from different factors, such as the need to not lose contact with students and also collaboration with colleagues and passion for their work. These factors positively influence the perceived competence in moving towards the Student, with the possibility to “re-imagine,” “re-build” oneself:

*But a real sailor can be seen in storms, right? So I had to ‘re-imagine, re-build myself’*. (PT1)

[Ma un vero marinaio si vede nelle tempeste: giusto? E allora mi sono dovuta “ri-immaginare, ri-costruirmi”.]

This change of attitude can already be seen in the titles of essays PT2, MT2, MT10, ST3, ST6, and ST13. For instance, ST3 states, “You never stop learning”:

*Thanks to the advice of some colleagues who had attended that famous course and were already using the Gsuite platform, after realizing that there was no time to lose, I started to ‘study’ how to carry out distance education. *(ST3)

[Grazie ai consigli di alcuni colleghi che avevano frequentato quel famoso corso e già usavano la piattaforma Gsuite, dopo aver realizzato che non c’era tempo da perdere, ho cominciato a ‘studiare’ come poter fare didattica a distanza.]

In this movement, positive changes of attitude are linked to a positive attitude towards teaching with technologies, in particular due to a good perceived competence. This emerges from the essays written by teachers (PT3, PT4, MT1, MT5, MT6, MT10, ST1, ST6, ST8, ST13, ST14, ST15, UT1, UT3) who claim to have a good relationship with technology already in their teaching practices preceding the lockdown. The resulting perceived competence seems to help overcome more quickly the initial discouragement and to provoke positive emotions towards having to get back in the game.

The teachers MT1, MT5, MT11, ST1, ST3, ST12, ST14, ST15, and UT3 find themselves in a new situation, putting themselves in the position of those who must/want to learn how to use new tools to manage it. However, different behaviors emerge from different teachers moving towards the Student. Some of them (MT2, MT3, MT5, MT9, MT10, ST6, ST14, ST15, UT3) refer acquiring technological skills, such as UT3:

*I am convinced that what we are experiencing can also be considered an opportunity to take contact and refine our knowledge of digital tools (even different software than those needed for the connection, such as viewers, calculators, calculation software, etc.) that will be very useful also in everyday teaching*. (UT3)

[Sono convinto che quella che stiamo vivendo può essere considerata anche un’opportunità di prendere contatto ed affinare le nostre conoscenze degli strumenti digitali (anche software diversi da quelli necessari al collegamento, come visualizzatori, calcolatrici, software di calcolo etc.) che potranno essere utilissimi anche nello svolgimento della didattica ordinaria.]

Other teachers (MT1, ST5, ST8, ST10) move towards the Student by trying to understand how to use technologies with educational objectives from the beginning, such as ST8:

*Personally, in order to continue the class work started in September and ended in early March, I thought it was appropriate to support the lessons in videoconference (with google-meet) i.e., synchronous, with lessons I’m going to record (with Screencast or matic) i.e., asynchronous*.

*[...]. Students can learn more easily if the calculation, the reasoning, takes place in a face-to-face setting: the Mathematica software allows me to write the procedure in a clear way, without the help of the graphic tablet.*

*In brief, I hope I get by*. (ST8)

[Personalmente, per continuare a svolgere il lavoro in classe iniziato nel mese di settembre e concluso agli inizi del mese di marzo, ho creduto opportuno di supportare le lezioni in videoconferenza (con google-meet) cioè sincrone, con lezioni che vado a registrare (con Screencast o matic) cioè asincrone.

[...] Gli alunni riescono ad apprendere più facilmente se il calcolo, il ragionamento, si svolge alla loro presenza: il software Mathematica mi consente di scrivere la procedura in modo chiaro, senza l’ausilio della tavoletta grafica. Insomma speriamo che me la cavo.]

The expression concluding ST8’s essay (*I hope I get by* [speriamo che me la cavo]) is a typical “student” expression in the geographical region in which the teacher belongs (taken from a well-known book in which a primary school teacher collected essays from students in a quite difficult socio-cultural context).

It is worthwhile to note that ST12 goes towards the Student in a very deep sense, almost as a researcher, who spends some days “looking for what was in the literature” about distance education:

*Personally, I spent the first three days of the school closing to research what was in the literature about Distance Education experiences. I immediately realised only one ‘thing’: it is not possible to move the teaching-learning practices in the classroom in distance education, you have to change more or less radically the content and teaching practices*. (ST12)

[Personalmente ho speso i primi tre giorni della chiusura delle scuole a cercare cosa ci fosse in letteratura a proposito di esperienze di Didattica A Distanza (DAD). Ho avuto subito un’unica ‘certezza’: non è possibile ‘migrare’ le pratiche di insegnamento-apprendimento in classe in modalità DAD, si devono cambiare più o meno radicalmente contenuti e pratiche didattiche.]

Positive attitudes towards technology seemingly favored the prevalence of a sense of challenge over that of bewilderment and confusion. Moreover, some essays suggest that, in addition to the movement towards the Student, there is also the movement towards the Author (such as ST13*: I don’t have the control of this work among peers…. How to promote it, even at a distance, so that it can encourage learning?* [Ma io non ho il controllo di questo lavoro tra pari…. Come promuoverlo, anche a distanza, in modo che possa favorire l’apprendimento?]).

### The teacher moves towards the Author

The radical change, the emotional impact, and the reflection on one’s own teaching led the teacher, in some cases, to describe what we interpreted as a movement of the teacher towards the Author and involving a change in the didactical transposition, due to the transition to distance education (i.e., ST15: *Distance education triggered me to reflect on my way of teaching* [La didattica a distanza ha innescato in me riflessioni sul mio modo di insegnare.])

The emotions and attitudes described in the essays are different and opposite: from the sense of bewilderment to the sense of freedom for no longer being subjected to institutional constraints (such as programs and assessments) that are seen as limits to one’s work.

The teacher goes towards the Author because she needs to design new teaching situations.

We identified two different ways to change. Some teachers try to make a didactical transposition trying to remain as similar as possible to what they did in a face-to-face setting, whereas some others feel the need for a change (others “suffer” this change). These views and beliefs are associated with different emotional dispositions. As an example, PT1 is one of the teachers expressing a positive change in her attitude, after the initial discouragement:

*I thought about a new ‘project/activity’ to be carried out online with the main aim of doing mathematics in a playful, joyful and engaging way, even if at distance. I then structured a class activity called ‘a team of detectives’*. (PT1)

[Ho pensato così ad un nuovo ‘progetto/iniziativa’ da svolgere on line con l’obiettivo prioritario di fare matematica in modo ludico, gioioso e coinvolgente anche se a distanza. Ho creato allora un’iniziativa di classe chiamata ‘una squadra di detective’.]

For PT3, PT7, MT2, ST15, UT1, and UT3, the new didactical transposition is an adaptation of the previous one. As an example:

*the attempt was to restart teaching as similar as possible to the previous one* (MT2)

[il tentativo era quello di riprendere una didattica più simile possibile a quella precedente.]

Nevertheless, the essays of PT5, MT8, MT10, ST4, ST5, ST6, ST12, and ST13 show the need to rethink and redefine a new didactic transposition. As an example:

*What I had to redefine in this first period is the idea of the lesson itself* (PT5)

[Ciò che più ho dovuto ridefinire in questo primo periodo è l’idea stessa di lezione.]

While at beginning the term “video lessons” is widely used to indicate an adaptation of face-to-face lessons used before the lockdown; in this case, as ST12 writes, video lessons have a different meaning:

*it is not possible to ‘migrate’ teaching and learning practices in the classroom in Distance Education mode, you have to change more or less radically the contents and teaching practices [...] Making them immediately part of my choice, I exclude that Distance Education coincides with video lessons, precisely because I do not believe that you can transpose the ‘physical’ class into a ‘virtual’ class. The video lesson will be an important but not exclusive moment*. (ST12)

[non è possibile ‘migrare’ le pratiche di insegnamento-apprendimento in classe in modalità DAD, si devono cambiare più o meno radicalmente contenuti e pratiche didattiche [...] Escludo, facendoli subito partecipi della mia scelta, che DAD coincida con videolezioni, proprio perché non credo che si possa trasporre la classe ‘fisica’ in una classe ‘virtuale’. La videolezione risulterà un momento importante ma non esclusivo.]

In some essays, the positive emotions in the movement towards the Author seem linked by a real “sense of liberation” from traditional teaching and anxiety for the syllabus:

*My first impact with distance education was a great joy for me, I was happy to be able to apply methodologies and tools that I could not always use in class. [...] I am focused exclusively on learning and not at all on completing programs, an anxiety that, unfortunately, I was experiencing even if I was aware of the mistake [...] there is room for knowledge and skills but above all for development and evaluation of competencies*. (ST6)

[Il primo impatto con la didattica a distanza è stato per me una grande gioia, ero contenta di poter applicare metodologie e strumenti che in classe non sempre riuscivo ad utilizzare. [...] sono concentrata esclusivamente sugli apprendimenti e per nulla sul completamento dei programmi, ansia che purtroppo vivevo anche se consapevole di sbagliare […] c’è spazio per conoscenze e abilità ma soprattutto sviluppo e valutazione di competenze.]

The reflection linked to the movement towards Author leads teachers to consider aspects of their own professional activity that may be new to them, as in the case of PT3:

*Distance education needs a preliminary phase of didactic design through the selection of contents, the identification of objectives and the contextualization of the didactic unit within the disciplinary program. A work of selection and organization of materials to be used, of activities to be proposed for education and curricular disciplines carried out in synchronous and asynchronous way*. (PT3)

[La DAD ha bisogno di una fase preliminare di progettazione didattica attraverso la selezione dei contenuti, l’individuazione degli obiettivi e la contestualizzazione dell’unità didattica all’interno del programma disciplinare. Un lavoro di selezione e organizzazione di materiali da utilizzare, di attività da proporre per educazioni e discipline curriculari somministrate in forma sincrona e asincrona.]

Here, PT3 refers to teaching processes that ought to always be taken into consideration yet are evidently new to him. The process, in this sense, only comes to the surface when PT3 loses some of her certainties.

### The teacher moves towards the Tutor

By looking into this movement, we commit to study the dynamics whereby unexpected and sudden change in teaching delivery influenced the teacher–student relationship. Many of the essays analyzed here focus on such mechanisms. PT2, MT7, ST1, ST2, ST14, and ST16 narrate how the first and very strong perceived need was not related to teaching/learning mathematics; rather, it was about making children feel close (the need to “not leave them alone,” as PT2 writes), behaving more than before (and in a different way) as an “adult reference point” for the students, in such a moment of general disorientation. The emotions emerging from the essays are very strong, both positive and negative, described with emphasis and with many lines of text, as the following excerpt shows:

*What is changed is that now it is no longer a question of homework or teaching, it is about being an adult reference and less a teacher. Having an appointment every day helps to sustain this situation made of fear, sometimes of sick parents, of flimsy internet connections, of crowded rooms, of embarrassment in showing one’s home intimacy and nostalgia of the classmates. [...] the only thing that I have managed to do till today I think is not having lost them but this has nothing to do with teaching*. (MT7)

[Cosa è cambiato allora? E’ cambiato che ora non si tratta più né di compiti né di didattica, si tratta anche di essere ancora più un adulto riferimento e meno insegnante. Il fatto di avere un appuntamento ogni giorno aiuta a sostenere questa situazione fatta di paura, talvolta di genitori ammalati, di internet che non va, di stanze affollate, di imbarazzo nel mostrare la propria intimità casalinga e di nostalgia dei compagni.[...] l’unica cosa che ad oggi sono riuscita a fare credo che sia il fatto di non averli persi ma ciò con la didattica non ha nulla a che fare.]

The narratives of PT5, MT9, MT11, ST2, ST7, ST13, ST17, and UT1 show new awareness of emotions, that perhaps were previously not so explicitly relevant:

*I realized I love my pupils very much*. (MT11)

[Mi sono accorta di volere molto bene ai miei ragazzi.]

This shift towards the Tutor is very evident in the essays of teachers of all levels, even in the ones from high school:

*We see a relationship in which the collaboration and the willingness to meet each other become the real drive to move forward and to build a functional future for us and for them*. (ST17)

[Vediamo formarsi una relazione in cui la collaborazione e la volontà di venirsi incontro diventano la vera forza per andare avanti e per costruire un futuro funzionale per noi e per loro.]

The shift furthermore entails a change in the relationship, an increase in confidence and trust:

*This activity, however, although tiring and expensive, brings me closer to my students, in some way it seems to me to ‘cuddle’ them, caress them one by one, in order to be able to follow and advise them [...] I must recognize that this anomalous situation has greatly increased the confidence and trust among us; I perceive the need they have to be reassured and guided and I realize that the trust they place in me has increased*. (ST2)

[Questa attività però, anche se faticosa e onerosa mi avvicina ai miei studenti, in un qualche modo mi sembra di ‘coccolarli’, accarezzarli uno per uno, per poterli seguire e consigliare [...] devo riconoscere che questa situazione anomala ha molto aumentato la confidenza e la fiducia tra noi; percepisco il bisogno che hanno di essere rassicurati e guidati e mi accorgo che è aumentata la fiducia che essi ripongono in me.]

In the essays of PT2, PT5, MT1, MT6, UT1, and MT2, this great need emerges also in the opposite direction, that is, the need of the teacher themselves to be tutors for their students, in such a particular moment:

*The emotional pain will be long, but being able to start again communicating with the students is a cure-all for me. It gives a purpose to my days*. (MT1)

[La sofferenza emotiva sarà lunga, ma potere riprendere la comunicazione con i ragazzi è per me un toccasana. Dà uno scopo alle mie giornate.]

This movement also includes a change in the teachers’ understanding of assessment. Distance as well the greater amount of data provided by technological equipment allows the teacher to have a holistic view of the students’ knowledge. In this sense, the teacher tends to make more extensive use of formative assessment and therefore dropping summative assessments. Indeed, it seems that MT1, MT3, MT5, MT8, MT10, ST3, ST4, ST6, ST8, and ST10 feel “free” to behave as tutors in relation to the assessment, not worrying about “grades” (MT3: *free from the bureaucratic trammels and cancer that afflicts our school: number grades.* [libero dalle pastoie burocratiche e dal cancro che affligge la nostra scuola: i voti in numero.]), focusing instead on feedback and formative assessment (MT1: […] *evaluation is still important, in a formative sense. Giving feedback to children is fundamental*. [la valutazione è comunque importante, in senso formativo. Dare un feedback ai ragazzi è fondamentale])*.*

A very recurring theme in teachers moving towards the Tutor, characterized by very strong and almost always negative emotions such as fear, worry, and anger, is that of the feeling of not being able to include or “keeping” children in difficulty. In the essays of PT3, MT1, MT7, MT8, MT12, ST1, ST8, and ST16, there is a constant fear that distance education may create or exacerbate inequalities due to different factors (socio-cultural context, learning difficulties, greater or lesser technological availability, greater or lesser family support).

MT8, whose essay narrates a very difficult social context, writes:

*In many families, the lack of digital skills and tools necessary for ‘distance education’ has widened the gap between social classes, undermining the essential value of universality that the right to study should have*. (MT8)

[In molte famiglie la mancanza di competenze e strumenti digitali necessari per la ‘didattica a distanza’ ha aumentato il gap tra le classi sociali minando l’imprescindibile valore di universalità che dovrebbe avere il diritto allo studio.]

Still very strong negative emotions emerge from MT12 and ST16:

*That’s why I’m angry at the slogan ‘Let’s leave no one behind’ [...] In lower socio-economic demographics, but also in the medium poor and difficult or simply heterogeneous ones, distance education often let the last ones remain last, therefore behind*. (MT12)

[Ecco perché mi fa rabbia lo slogan: ‘Non lasciamo indietro nessuno’ [...] Nei quartieri di frontiera, ma anche mediamente poveri e difficili, o semplicemente eterogenei, con la Dad succede che gli ultimi rimangono ultimi, perciò indietro.]

*Needless to say, distance education has unfortunately accentuated the enormous social gap that already exists among students of our schools*. (ST16)

[Inutile dire poi che la didattica a distanza ha purtroppo accentuato l’enorme divario sociale già esistente tra gli alunni delle nostre scuole. ]

Just two teachers (PT5, MT12) think with fear at the moment when they will “return back to class”:

*on the day of a hypothetical return to class, the differences between students will be enormous [...] Distance education is not inclusive by nature, and in my humble opinion, it can never be*. (PT5)

[il giorno di un ipotetico rientro le diversità dovute al nucleo familiare saranno enormi. [...] La didattica a distanza non è inclusiva di natura, e a mio modesto parere, non potrà mai esserlo.]

### The teacher moves towards the Mathematics

The movement towards the Mathematics is determined by the movement towards the other vertices of the tetrahedron. This movement is activated by the need of the teacher to revise her previous didactic transposition to be effective in this new context, which requires a reflection on Mathematics.

As an example, ST4 illustrates her view of mathematics (see the terms “unnecessary” and “baroque” in the excerpt below), suggesting that the new context allows her to manage the teaching/learning process in a way coherent with her views. Moreover, ST4 notes that this coherence between the view and the didactic action allows her to achieve the intended educational goals: the essay reveals that she is perceiving such experience as entirely new:

*I am experiencing that these didactic goals*^4^*are achievable without complicating life with unnecessary algebraic ‘baroque’ manipulations (for example solving equations by means of 15 simplification steps before arriving at the ‘final’ equation itself ...). The students are reacting well. Better than they did pre-coronavirus*. (ST4)

[Sto facendo esperienza che questi obiettivi sono raggiungibili senza complicarsi la vita con inutili ‘baroccheggiamenti’ algebrici (esempio con equazioni da semplificare in 15 passaggi prima di arrivare all’equazione ‘finale’ vera e propria…). I ragazzi stanno rispondendo bene. Meglio di quanto fatto pre-coronavirus.]

For this new transposition, the teacher needs to pass through the Student (she reflects on the technologies to use in order to carry out the new transposition) and the Author (she reflects on how to realize a new didactic transposition). These movements reveal how the view of mathematics and its teaching ultimately influences the emotional dispositions of teachers.

On the one hand, for UT1 and UT2, the fear of failing to keep their own vision or a low perceived competence influences the emergence of negative emotions, especially at the beginning. On the other hand, PT1 and ST12 reconsider mathematics as a meeting place, as an occasion even more important than before to strengthen relations with students, rather than a knowledge to be taught. For PT1, for instance, the creation of a comfortable environment can support students from an emotional point of view and support their learning process too:

*And all of this has to do with mathematics? I studied and lived in my own skin and in that of the ‘fragile’ children I meet, that emotions are fundamental in every learning process, and also in numbers, and even in this difficult moment! I will continue, with my wizard hat, to meet the pupils and they, perhaps, will feel as a friend, which encourages them because ‘learning is a fantastic adventure’! *(PT1)

[E tutto questo che contatto ha con la matematica? Io ho studiato e vissuto sulla mia pelle e su quella dei bambini ‘fragili’ che incontro, che le emozioni sono fondamentali in ogni processo di apprendimento, e anche nei numeri, e anche in questo momento così difficile! Io continuerò, con il mio cappello da maga ad incontrare gli alunni e loro, forse, avvertiranno una presenza, amica, che li incoraggia perché ‘apprendere è un’avventura fantastica’!]

The excerpts of PT3, MT3, MT4, MT10, MT11, ST6, and ST7 highlight how the teacher moves towards the Mathematics feeling the need to pass via the Author:

*When it was necessary to introduce an absolutely new [mathematics] topic and trying to make it understood, I found myself in great difficulty. I found a setting that seems to work. *(PT3)

[Nel momento in cui, invece, si trattava di introdurre un argomento del tutto nuovo cercando di farlo comprendere, mi sono trovata molto in difficoltà. Ho individuato un’impostazione che mi sembra funzionare.]

The teachers PT5, MT9, MT2, MT4, MT6, MT7, ST3, ST5, ST6, and ST10, instead, move towards the Mathematics via the Tutor. For example, PT5 reflects on the importance of the feedback to maintain contact with students:

*Then I had to introduce new arguments, so I introduced multiplication using all the different possible strategies [...] But then the big problem of feedback arose [...] only the relationship guarantees useful feedback to me and the students because it allows me to go further and reach that emotional and motivational level, so important for learning [...] A distance education program can’t take care of all that. The term ‘distance’ itself says so.* (PT5)

[Mi è stato poi però richiesto di introdurre argomenti nuovi, quindi ho introdotto la moltiplicazione utilizzando tutte le diverse strategie possibili [...] Ma poi si è posto il grande problema del feedback. [...] solo la relazione mi permette di garantire feedback utili a me insegnante e agli alunni, perché mi permette di andare oltre e raggiungere anche quel livello affettivo e motivazionale, così importante per l’apprendimento [...] A tutto questo una didattica a distanza non può per natura sopperire. Lo dice il termine stesso ‘distanza’.]

## Discussion and conclusions

This study is based on the assumption that the researchers interpret phenomena by means of the narrators’ words, producing sense and understanding (Bell, 2002). Summing up, we have collected essays where teachers chose what to narrate about their feelings in the pandemic situation and then their perception of the changes, their reflections, and their emotions related to themselves, to students, to mathematics education, and to distance education.

The analysis of the essays was carried out through a double theoretical lens, the e-learning tetrahedron model (Albano, 2017), and the teachers’ attitude model (Coppola et al., 2012). These two perspectives allowed us to identify elements of interest and their relations in order to analyze the process of change and the way in which teachers have experienced and perceived it.

First of all, in the collected data, we can identify two temporal periods: a first period of bewilderment (e.g., ST14: *Carrol’s Alice, into a space with an unknown and deforming topology* [Alice di Carroll, in un spazio dalla topologia sconosciuta e deformante]) and a second one of reflection and elaboration (e.g., ST15: *Distance education has triggered in me reflections on my way of teaching, on my (poor) flexibility, on the best way to transmit and involve* [La didattica a distanza ha innescato in me riflessioni sul mio modo di insegnare, sulla mia (scarsa) flessibilità, sul modo migliore di trasmettere e coinvolgere]). This second period is generally perceived differently, as if the situation was less negative than expected before, and a change in the attitude seems to emerge, in particular with high school teachers, together with the movements towards the vertices of the tetrahedron.

The first period began at a well-fixed time when something dramatic happens, the media announcement of the lockdown. It is a “watershed,” a *turning point* (Bruner, 1990) after which nothing has been as before. The impact of the lockdown on teachers has been disruptive. Essentially, very strong emotions arising from the closure of the schools are described in all the essays, where this moment is narrated with great emphasis together with the space–time description of the context (the weather, the place where the teacher was while learning the news, etc.). These strong emotions affect both the professional and the personal sphere. The former concerns the lack of possibility to carry out the normal didactic activity, with the discomfort of having to manage one’s own professional activity without feeling to have the tools to do so (that is, with a low perceived competence). The latter sphere concerns the personal distress coming from the absence of “contact” with the students.

The description of the second period (of reflection) starts very often with the description of distance education in negative terms: “it cannot,” “it is not,” “it is impossible”. Generally, it is just what is missing that triggers a reflection, from which it emerges that the teachers take into consideration new (for them) perspectives on various functions of the teacher, on educational objectives, on mathematics, and on mathematics education. Interestingly, the teachers refer to this situation in terms of opportunities. For example, a primary teacher (PT6) writes that “the tempting opportunity that this situation has offered us is the possibility to rethink and re-evaluate our school microworld” [l’occasione ghiotta che ci ha offerto questa situazione è la possibilità di ripensare e rivalutare il nostro micromondo della scuola.]. Similarly, a secondary school teacher (ST10) writes*: *“I am firmly convinced […] that this change in which we have been catapulted should be taken as an opportunity” [sono fermamente convinta […] che questo cambiamento in cui siamo stati catapultati vada colto come un’opportunità.])*.*

Actually, what teachers report concerns educational issues widely discussed in the literature in mathematics education such as educational goals, design of learning activities, assessment, and so on. Nevertheless, these issues coming out from reflection appear as completely new for these teachers. In other words, they seem to discover some key aspects of the didactic system in which they are embedded, thanks to the disruption of the didactic system itself.

Generally speaking, the traumatic change in the educational setting plunged teachers into an unexpected and unthinkable world where the teachers become aware that the didactic system has to be reconstructed. Therefore, totally different educational worlds are imagined: a school where the summative assessment disappears; a school where mathematics is not a set of formula and procedure; a school where the teachers design activities to promote reasoning and, more generally, competency-oriented activities; and a school where technology is really integrated in teachers’ and students’ usual practice. These imagined educational worlds are new (for the teachers) due to a different role of the teacher, a different teacher’s attitude towards mathematics, and its teaching and a different epistemology of mathematics. We think that it is relevant that teachers take into consideration different *possible worlds* (in the sense of Bruner, 1986) that can assume an important role in the development and in the diffusion of a culture of mathematics education, even if these *possible worlds* will not (fully) become *actual worlds*.

The construction of possible worlds can foster different teachers’ attitude towards their professional development, enabling an alignment and a dialogue between the new (for them) issues described above and the related development and results in mathematics education research. This alignment can have a deep impact on teachers’ education that has to be further explored.

However, future research is needed to investigate the teachers’ reflection and the teachers’ movement in the tetrahedron along the persistent pandemic. New periods could be identified besides the two temporal periods above described, and it would be interesting to inquire about the role that these new periods can have in the actualization of the *possible worlds*.

Finally, from the point of view of the elaboration of the theoretical framework, two specific issues remain open. The first concerns the role of technology in the tetrahedron. It appears from the essays as a means for approaching teachers and students within the didactic system: this approaching is bi-directional, since it is not only the teacher who is in charge of institutionalizing students’ knowledge, but she is recognized by the student as the one who takes care of the students’ learning and works to this aim. The physical distance prompts teachers’ reflection on the role of the contact between teacher and students, and this suggests somehow a relation between external and internal spheres of the tetrahedron model that need further exploration.

The second unresolved theoretical issue concerns the relations between the theoretical constructs used in the analysis. The teachers’ attitude model (Coppola et al., 2012) and the e-learning tetrahedron model (Albano, 2017) have allowed an analysis from two perspectives that reciprocally enriched each other. On the one hand, the e-learning tetrahedron does not take into account the affective aspects; on the other, the attitude model does not pay attention to technology or didactical elements, including design and tutoring. The analysis process and the findings show that a deeper theoretical integration in terms of networking is possible (Prediger et al., 2008) and further studies will be needed in this direction.

## Footnotes

1 ALICE project (Adaptive Learning via Intuitive/Interactive, Collaborative and Emotional systems, Collaborative project, FP-ICT-2009–5).

2 Throughout the paper, we will indicate in italics the translation of the original Italian excerpts reported not in italics.

3 Upper and lower case as in the original essay.

4 Here, the teacher refers to items from mathematics syllabus, such as solving equations/inequations and study of a function.
